# High dietary and lifestyle inflammatory scores are associated with increased risk of chronic kidney disease in Iranian adults

**DOI:** 10.1186/s12937-023-00835-y

**Published:** 2023-01-07

**Authors:** Hossein Farhadnejad, Farshad Teymoori, Mitra Kazemi Jahromi, Ebrahim Mokhtari, Golaleh Asghari, Parvin Mirmiran, Fereidoun Azizi

**Affiliations:** 1 Nutrition and Endocrine Research Center, Research Institute for Endocrine Sciences, Shahid Beheshti University of Medical Sciences, Tehran, Iran; 2 Department of Clinical Nutrition and Dietetics, Faculty of Nutrition Sciences and Food Technology, National Nutrition and Food Technology Research Institute, Shahid Beheshti University of Medical Sciences, Tehran, Iran; 3 Department of Nutrition, School of Public Health, University of Medical Sciences, Tehran, Iran; 4 Endocrinology and Metabolism Research Center, Hormozgan University of Medical Sciences, Bandar Abbas, Iran; 5 Endocrine Research Center, Research Institute for Endocrine Sciences, Shahid Beheshti University of Medical Sciences, Tehran, Iran

**Keywords:** Dietary pattern, Lifestyle, Inflammation, Chronic kidney diseases, Adults

## Abstract

**Background:**

Systemic inflammation can be the initiator in developing chronic diseases that may be affected by the lifestyle and diet of individuals. In the current study, we aimed to assess the association of the inflammatory potential of diet and lifestyle, determined by the food-based index of dietary inflammatory potential (FBDI), dietary inflammation score (DIS), and lifestyle inflammation score (LIS), with risk of chronic kidney disease(CKD) in Iranian adults.

**Methods:**

A total of 6044 CKD-free individuals aged ≥ 18 years, were recruited from among participants of the Tehran Lipid and Glucose Study(surveys 3 and 4) and followed a mean of 6.03 years(follow-up rate:94.95%). Data on dietary intakes were determined using a food frequency questionnaire. The inflammatory potential of diet and lifestyle were determined based on three indices, including FBDI, DIS, and LIS. Using the National Kidney Foundation guidelines, we defined CKD as eGFR < 60 mL/min/1.73 m^2^.

**Results:**

Mean ± SD age of the study population(54.3% women) was 37.8 ± 12.8 years. We identified 1216(20.1%) new cases of CKD during the 6.03 years of follow-up (46,889.8 person-years). In the multivariable-adjusted model, the risk of CKD incident is increased across quartiles of FBDI (HR = 1.21;95%CI:1.03–1.42, P_trend_:0.014) and LIS (HR = 1.28;95%CI:1.07–1.55,P_trend_:0.006). However, no significant relationship was observed between the higher DIS score and CKD risk.

**Conclusion:**

Our findings showed that a higher inflammatory potential of diet and lifestyle, characterized by a higher score of FBDI and LIS, was related to increased incidence of CKD, while no significant relationship was reported between the DIS score and CKD incident.

## Background

Chronic kidney disease (CKD) is one of the major worldwide health concerns [1], which has a significant contribution to the global burden of disease by incrementing cardiovascular disease (CVD) and mortality risk [2, 3]. According to the international guidelines, the existence of structural and functional damage, albuminuria, and glomerular filtration rate (GFR) below 60 ml/min/1.73 m^2^ considered as the definition of CKD if these circumstances have been lasted for at least three months and have not been caused by other causes [4]. Based on the latest Global Burden of Disease report in 2017, the CKD prevalence worldwide was estimated at 9.1%. This prevalence increased by about 29.3% in all ages compared to 1990 [1]. Recent national data has also reported that the overall CKD prevalence in Iranian adults was more than 10% [4]. An unhealthy lifestyle and poor diet, alongside some multifactorial abnormalities such as hypertension, obesity, and diabetes, are readily treatable major risk factors for the causes of CKD [5, 6].

Low-grade systemic inflammation is of considerable importance in the pathophysiology of CKD [7], which can significantly increase the morbidity and mortality associated with chronic nephropathy [8]. Studies have shown that an unhealthy lifestyle is an important risk factor for elevated systemic inflammation [9]. Also, the individual relationship of main lifestyle determinants, including diet, physical activity, body weight, and smoking with inflammation has previously been well established and shown that each one is an important determinant of predicting the inflammation status, per se [10–13]. However, due to the interactions of these factors with each other, it makes sense that the simultaneous study of the combined role of these factors significantly improves the ability to predict the outcome.

Accordingly, in recent years, the diet inflammation score (DIS) and lifestyle inflammation score (LIS) has been introduced by Byrd et al. to evaluate the ability of diet and other lifestyle-related factors to aggravate or modulate systemic inflammation levels in the body [14]. Indeed, DIS and LIS determine the collective contributions of lifestyle and diet exposures to systemic inflammation. Another index recently developed by Na et al. in the Korean population is food-based dietary inflammatory potential (FBDI), which uses the hs-CRP biomarker as a response variable to determine the inflammatory effects of food groups [15]. Although, to the best of our knowledge, no study has yet assessed the association of DIS, LIS, and FBDI scores with CKD risk, the relationship of these lifestyle and diet inflammatory scores with CKD-related disorders such as type 2 diabetes (T2D) and metabolic syndrome (MetS) and other chronic diseases has been investigated previously [15–20]. Two studies showed that higher DIS and LIS scores are associated with an increased risk of T2D and MetS [17, 20]. Other studies have also linked DIS and LIS scores to CVD and cancers [16, 18, 19]. Limited studies have examined the performance of FBDI [15, 21]. Na et al. showed that an increased FBDI score was associated with a higher prevalence of MetS [15].

Although there are no data on the clear kidney-related effect of lifestyle and diet inflammatory score in subjects with healthy renal function, the observed association of these inflammatory indices with the risk of T2D, MetS, and CVD that are closely related to renal disease, suggests a possible link between these inflammatory indices and the CKD risk. Therefore, this study aimed to investigate the relationship between DIS, LIS, and FBDI scores and CKD risk among Iranian adults.

### Materials and Methods

#### Study participants

The present study was performed in the framework of the Tehran Lipid and Glucose Study (TLGS), a population-based cohort study conducted to examine the chronic diseases risk factors among a representative urban population of Tehran, including 15 005 participants aged ≥ 3 years [19]. The first survey of TLGS was initiated in March 1999, and data collection conducted prospectively at 3-year intervals is ongoing. The baseline survey was a cross-sectional study performed from 1999 to 2001, and surveys II (2002–2005), III (2006–2008), IV (2009–2011), V (2012–2015), and VI (2015–2018) were prospective follow-up surveys. The details of the TLGS have been reported previously [22]. In the third survey of the TLGS (2006–08), of 12 523 participants, 3568 were randomly selected for dietary assessment. Also, in the fourth survey (2009–2011), of 12 523 participants, 7956 randomly selected subjects agreed to complete dietary assessment.

**Fig. 1:**
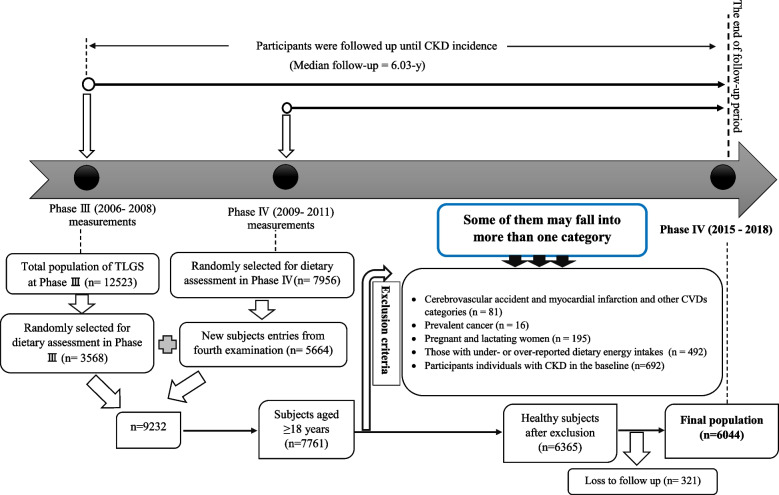
Timeline of the current study

For the current study, participants aged ≥ 18 years, with complete nutritional information on the third examination of TLGS and the new entries participants in the fourth examination, which was 7761, were included. Individuals with cardiovascular accidents and myocardial infarction (n = 81), and prevalent cancer (n = 16) were excluded. Also, the pregnant and lactating women (n = 195), those with under- or over-reported dietary energy intakes (out of the range 800–4200 kcal/d) (n = 492), and individuals with CKD in the baseline (n = 692) were excluded; some of them may fell into more than one category. Of 6365 participants at baseline, who were followed-up to sixth survey of TLGS (2015-2018), 321 were lost to follow-up, and 6044 remained for final analysis (follow-up rate: 94.95%) (Fig. 1).

#### Physical activity assessment

The individual’s physical activity data were collected by a modifiable activity questionnaire (MAQ), previously modified and validated among Iranian adults [23]. Individuals were asked to report and identify the frequency and time spent on activities of light, moderate, hard, and very hard intensity, over the past year, based on a list of common activities of daily life; we reported the total physical activity of each participant as metabolic equivalent/hours per week (Met.h.wk).

#### Demographic and anthropometric assessment

Trained and expert interviewers used a standard questionnaire to collect study population data on socio-demographic characteristics, including age (years), sex, education level (high school and diploma, academic education), smoking habit, medical history, and medications at baseline. We used a standardized mercury sphygmomanometer with an accuracy of two mmHg to measure the systolic blood pressure (SBP) and diastolic blood pressure (DBP). All blood pressure measurement was performed for each participant twice on the right arm with a minimum interval of 30 s after a 15-min rest sitting on a chair; we considered the mean of the two measurements to be the participants' blood pressure. We measured participants' body weight using a digital scale (Seca 881, Germany) to the nearest 100 g while the participants were in light clothes and without shoes. Height was measured by a stadiometer in a standing position without shoes and recorded to the nearest 0.5 cm. BMI was computed as weight (kg) divided by the height squared (m^2^).

#### Biochemical measurements

The biochemical variables, including fasting blood glucose (FPG), 2-h blood glucose, and serum creatinine were measured in participants. Based on the standard protocol, participants' blood samples were taken after 12–14 h of overnight fasting in a sitting position and centrifuged within 30–45 min of collection. We performed all blood analyses at the TLGS research laboratory and used the Selectra 2 auto-analyzer (Vital Scientific, Spankeren, The Netherlands) to analyze the samples. FPG was determined using an enzymatic colorimetric method with glucose oxidase. Both inter-and intra-assay coefficient variations were 2.2% for FPG. For the oral glucose tolerance test, 82.5 g of glucose monohydrate solution (equivalent to 75 g anhydrous glucose) was administered orally to participants aged > 20 years. A second blood sample was taken 2-h after glucose ingestion. Serum creatinine concentration was measured based on the standard colorimetric Jaffe Kinetic reaction method. Both intra- and inter-assay CVs were < 3.1%. We performed all analyses using commercial kits (Pars Azmoon Inc., Tehran, Iran).

#### Definitions

Hypertension (HTN) was determined in the study population based on SBP/DBP ≥ 140/90 mm Hg for individuals aged < 60 years and SBP/DBP ≥ 150/90 mm Hg for those aged ≥ 60 years or using current antihypertensive medication [24]. The criteria of the American Diabetes Association (ADA) were used to determine T2D in participants according to the following criteria: FPG ≥ 126 mg/dl or 2-h post 75-g glucose load ≥ 200 mg/dl or current blood glucose-lowering medications [25]. The Epidemiology Collaboration (EPI) equation formula, as described by Levey et al. [26], was used to calculate eGFR in participants. We expressed the eGFR was in ml/min/1.73m^2^ of body surface area. CKD was defined based on participants’ eGFR levels using the national kidney foundation guidelines as follows: eGFR ≥ 60 ml/min/1.73m^2^ as not having CKD and eGFR < 60 ml/min/1.73m^2^ as having CKD.

#### Dietary intake assessment

A valid and reliable 168-item semi-quantitative food frequency questionnaire (FFQ) with standard serving sizes was used to determine the participant’s dietary intake data in the last year [27]. Expert nutritionists with at least five years’ experience in TLGS asked individuals to report the frequency of their intakes for each food item on a daily, weekly, monthly, or yearly basis; portion sizes of consumed foods, reported in household measures, were then converted to daily grams of food intake. Considering that the Iranian Food Composition Table (FCT) is incomplete and has limited data on the nutrient content of raw foods and beverages, we used the United States Department of Agriculture (USDA) FCT. However, the Iranian FCT was used for local food items not listed in the USDA FCT.

#### Calculation of scores

DIS score were calculated based on the Byrd et al. study [14] based on 19 food components. High-sensitivity C-reactive protein and interleukins (interleukin-6, interleukin-8, and interleukin-10) were considered as response variables for the development of this inflammatory dietary index. According to each food item's effect on the inflammatory indicators levels, each item was assigned a specific weight, which could be positive or negative. DIS encompasses originally included leafy greens and cruciferous vegetables, tomatoes, apples and berries, deep yellow or orange vegetables and fruit, other fruits, and natural fruit juices, other vegetables, legumes, fish, poultry, red and organ meats, processed meats, added sugars, high-fat dairy, low-fat dairy and tea, nuts, other fats, refined grains, starchy vegetables, and supplements intake. However, supplement intake was excluded from the calculation of DIS in this study due to the lack of information in our dataset; then, we computed the overall score with 18 food groups. To compute the DIS score, each food item was multiplied by its specific weight (explained in Byrd et al. study) to determine the weighted values of each item. The weighted values were then standardized using the Z-score (to a mean of zero and SD of 1). Finally, all the items' standardized weighted values were summed to calculate the DIS score for participants [14].

LIS score was calculated using the physical activity, BMI, and smoking status data based on the Byrd et al. study [14]. Due to religious and legal restrictions in the Iranian population, alcohol is not consumed, or its consumption is not reported, so we did not consider alcohol consumption to calculate the LIS score. First, a dummy variable was created from each component and then multiplied for proposed regression coefficients [14]: physical activity was categorized into tertiles, and participants in the first, second, and third tertiles gave 0.0, -0.18, and -0.41, respectively. Participants were categorized into average weight (BMI < 25), overweight (25 ≤ BMI < 30), and obese (BMI ≥ 30); and then respectively received 0.0, 0.89, and 1.57 scores. Also, the proposed regression coefficients for smokers vs. non-smokers were 0.50 vs. 0.0, which were assigned. Finally, all the weighted values were summed to calculate the LIS score.

FBDI score was calculated according to the Na et al*.* study [15]. They used the Spearman correlation analysis and multiple regression for selecting food components and creating a formula to determine the FBDI score. In the Na et al*.* study, the spearman correlation analysis between log hs-CRP and the 51 food components was determined to select food components (ten dietary components) that were considered a significant correlation. Finally, FBDI was developed based on ten selected food items: mixed coffee and sweetened drinks, white rice, green vegetables, eggs, citrus, legumes, red fruits, beef, bread and wheat flour, and nuts. To calculate the FBDI score, the intakes of each of the mentioned food groups were multiplied by its specific applied value (weight). Finally, all ten weighted intake values were summed to form an overall FBDI score [15].

#### Statistical analyses

We used the Statistical Package for Social Sciences (Version 20.0; SPSS, Chicago, IL) to perform all statistical analyses. The Kolmogorov–Smirnov test and histogram chart were used to assess the normality of variables. Baseline characteristics of the participants are expressed as the mean ± SD or median (25–75 interquartile) for quantitative variables and percentages for qualitative variables. Individuals were classified according to FBDI and DIS quartiles cut-off points. Chi-square and linear regression were used to test for trends of categorical and continuous variables across quartiles of FBDI and DIS (as the median value in each quartile), respectively. Multivariable Cox regression models were used with CKD as the dependent variable and FBDI, DIS, and LIS as independent variables to estimate the risk of incident outcomes. We reported the hazard ratios (HRs) and 95% confidence intervals (CIs). The first quartile of each above-mentioned lifestyle and diet inflammatory score was considered the reference group. The multivariable model was adjusted for potential confounding factors, including age, sex, educational level, daily energy intake, hypertension, type 2 diabetes, BMI (adjusted for FBDI and DIS), smoking (adjusted for FBDI and DIS), physical activity (adjusted for FBDI and DIS), and baseline eGFR level. The proportional hazards assumption was checked using a log–log plot, and the assumption was satisfied (lines in the plots were parallel). P-values < 0.05 were considered to be statistically significant.

**Fig. 2:**
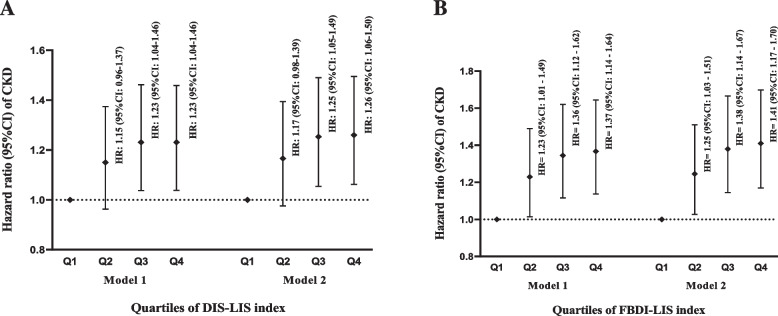
(**A-B**) The hazard ratios (HRs) and 95% confidence intervals (CIs) of chronic kidney disease across tertiles of DIS-LIS (A) and FBDI-LIS (B) based on model 1(adjusted for age and sex) and model 2 (adjusted for age, sex, energy intake, education level, baseline eGFR, hypertension, and type 2 diabetes)

In the current study, as additional analysis, we assessed the combined role of dietary inflammatory scores (FBDI or DIS) along with lifestyle inflammatory score (LIS) in predicting the risk of CKD incident; In order to perform this analysis, the total of DIS Z-score or FBDI Z-score with LIS Z-score (determined by using the SPSS software) were summed for all participants, and two new inflammatory scores including DIS-LIS and FBDI-LIS were determined for them. Then, individuals were divided into quartiles based on their scores for DIS-LIS and FBDI-LIS. Finally, the risk of CKD incident was determined for participants across the quartiles of these new inflammatory scores (Fig. 2A-B).

## Results

The mean ± SD age and BMI of study participants (54.3% females) were 37.8 ± 12.8 and 26.8 ± 4.7, respectively. The median (IQR) FBDI, DIS, and LIS in our participants were 4.40 (-3.85, 14.22), 0.12 (-0.84, 0.93), and 0.71 (0.00, 0.98), respectively. During the 6.03 years of follow-up, 1216 incident cases (20.1%) of CKD were reported (the incidence rate = 260 per 10.000 person-years) among all participants. Considering that in this study, after 6.03 years of follow-up, part of the initial population was excluded from the study for various reasons, therefore, we compared the baseline data on socio-demographic, anthropometric, biochemical, and clinical characteristics among the participants included in the final analysis and those excluded, and our results showed no significant difference (data not shown).

We showed individuals' baseline characteristics according to the quartile of FBDI score in Table 1. Individuals in the highest FBDI score quartile were more likely to be male, high smoked, and have higher education level and creatinine level than those in the lowest quartile of FBDI (P < 0.05). Dietary intakes of total fats (as a % of energy), sweetened drinks, white rice, and beef significantly increased across quartiles of FBDI score (P < 0.05). However, the intakes of total energy, carbohydrate (as a % of energy), protein (as a % of energy), green vegetables, eggs, citrus fruits, legumes, red fruits, bread wheat flour, and nuts were decreased across quartiles of this score (P < 0.001).

**Table 1:** Baseline characteristics of participants according to quartiles (Q) of the food-based dietary index of inflammatory potential

Variables	Food-based dietary index of inflammatory potential				P trend
	Q1 *n* = 1511	Q2 *n* = 1511	Q3 *n* = 1511	Q4 *n* = 1511	
FBDI score, median (interquartile)	-3.96 (-5.73, -2.72)	0.20 (-0.69, 1.08)	4.08 (2.94, 5.21)	10.69 (8.48, 14.98)	< 0.001
**Demographic, anthropometric, and other data**^**a**^					
Age (years)	38.7 ± 13.4	36.5 ± 12.6	37.2 ± 12.9	38.9 ± 12.1	0.194
Men (%)	39.6	42.4	47.5	53.2	< 0.001
Body mass index (kg/m2)	27.2 ± 4.8	26.6 ± 4.9	26.5 ± 4.5	26.8 ± 4.6	0.103
Smoking (%)	8.2	10.3	13.0	18.0	< 0.001
Physical activity (MET/hour/week)	62.5 (23.8 – 103.7)	63.6 (22.1 – 105.2)	64.1 (23.8 – 105.9)	71.4 (26.7 – 108.9)	0.086
Academic education, (%)	20.3	22.7	25.5	28.3	< 0.001
Type 2 diabetes (%)	4.8	3.7	3.7	4.4	0.353
Hypertension (%)	10.8	8.2	10.0	8.3	0.090
**Clinical data**^**b**^					
Creatinine (mg/dl)	1.02 ± 0.11	1.02 ± 0.11	1.02 ± 0.11	1.03 ± 0.11	0.028
Glomerular filtration rate (mL/min/1.73 m2)	80.0 ± 9.8	80.2 ± 9.8	79.7 ± 9.8	79.4 ± 9.8	0.116
**Nutrient Intake**^c^					
Energy(Kcal/d)	2565 (2530—2599)	2409 (2374—2443)	2344 (2310—2379)	2092 (2057—2126)	< 0.001
Carbohydrate(% of energy)	60.1 (59.7 – 60.5)	57.5 (57.1 – 57.9)	57.0 (56.6 – 57.4)	58.4 (58.0 – 58.8)	< 0.001
Protein(% of energy)	15.2 (14.9 – 15.5)	14.6 (14.3 – 14.9)	14.1 (13.8 – 14.4)	13.9 (13.6 – 14.2)	< 0.001
Fat(% of energy)	29.8 (29.1 – 30.4)	31.0 (30.3 – 31.6)	31.5 (30.9 – 32.2)	30.3 (29.7 – 31.0)	0.001
**FBDI components**^c^					
Sweetened drinks (g/d)	317 (293—340)	474 (449—499)	708 (681—735)	1212 (1184—1239)	< 0.001
White rice (g/d)	180 (173—188)	226 (218—234)	285 (250—266)	279 (271—287)	< 0.001
Green vegetables (g/d)	165 (160—170)	124 (119—129)	110 – (106—115)	97.4 (92.8—102)	< 0.001
Eggs (g/d)	17.2 (16.4 – 18.0)	15.1 (14.3 – 15.9)	14.3 (13.6 – 15.1)	12.9 (12.1 – 13.7)	< 0.001
Citrus Fruits (g/d)	172 (167—178)	112 (106—117)	91.2 (86.6 – 96.8)	77.3 (71.7 – 82.9)	< 0.001
Legumes (g/d)	49.1 (47.0 – 51.1)	35.3 (33.3 – 37.4)	30.4 (28.4 – 32.5)	24.8 (22.8 – 26.9)	< 0.001
Red fruits (g/d)	132 (127—138)	83.6 (78.1 – 89.1)	71.9 (66.3 – 77.4)	60.3 (54.8 – 65.9)	< 0.001
Beef (g/d)	15.0 (14.1 – 16.0)	17.7 (16.8 – 18.7)	19.1 (18.1 – 20.0)	17.8 (16.9 – 18.7)	< 0.001
Bread wheat flour (g/d)	254 (248—260)	204 (198—210)	181 (175—187)	148 (142—154)	< 0.001
Nuts (g/d)	10.6 (10.0 – 11.2)	7.7 (7.0—8.3)	7.0 (6.3 – 7.6)	6.0 (5.3 – 6.6)	< 0.001

^a^Data were represented as mean ± SD, or median (IQR 25–75) for continuous variables and percent for categorical variables. Chi-square and linear regression were used to test the trend of continuous and categorical variables across quartiles of the FBDI (as the median value in each quartile), respectively

^b^Data were represented as mean ± SD computed using univariate analysis adjusted for age and sex across quartiles of FBDI. P-value of the univariate analysis reported as P for trend

^c^Data were represented as mean (95%CI) computed using univariate analysis adjusted for age and sex across quartiles of FBDI. *P*-value of the univariate analysis reported as P for trend

The characteristics of participants according to the quartiles of DIS score at baseline are also reported in Table 2. Participants in the highest DIS score quartile were more likely to be male, younger, high smoked, and had lower academic education and percentage of T2DM and HTN than those in the lowest quartile of DIS (P < 0.05). Also, the level of physical activity was increased significantly across DIS score quartiles, whereas the mean BMI was decreased (P < 0.01). Furthermore, participants in the highest quartile of DIS score had higher intakes of total fat, red and processed meat, added sugar, and refined grains and starchy vegetables, but lower intakes of energy, carbohydrates, proteins, leafy greens and cruciferous vegetables, apples and berries, deep yellow or orange vegetables and fruit, other fruits and real fruit juices, other vegetables, legumes, nuts, fish, poultry, dairy products, and coffee and tea compared to those in the lowest quartile of DIS.

**Table 2:** Baseline characteristics of participants according to quartiles (Q) of the dietary inflammatory score

Variables	Dietary inflammatory score				P trend
	Q1 *n* = 1511	Q2 *n* = 1511	Q3 *n* = 1511	Q4 *n* = 1511	
**Demographic, anthropometrics, and other data**^**a**^					
DIS score, median (interquartile)	-0.73 (-1.07, -0.50)	-0.14 (-0.25, -0.03)	0.24 (0.15, 0.35)	0.65 (0.50, 0.83)	< 0.001
Age (years)	41.4 ± 12.7	39.1 ± 12.6	37.5 ± 12.5	33.4 ± 12.0	< 0.001
Men (%)	34.5	42.5	50.6	55.0	< 0.001
Body mass index (kg/m2)	27.6 ± 4.6	27.1 ± 4.6	26.5 ± 4.8	25.8 ± 4.6	< 0.001
Smoking (%)	9.8	11.7	13.1	15.0	< 0.001
Physical activity (MET/hour/week)	62.1 (22.3 – 104.1)	62.0 (22.3 – 102.5)	69.2 (29.8 – 107.4)	68.9 (23.8 – 108.7)	0.009
Academic education, N (%)	23.0	25.3	26.7	21.8	0.008
Type 2 diabetes (%)	5.5	4.5	4.1	2.5	< 0.001
Hypertension (%)	11.8	9.2	9.1	7.3	0.001
**Clinical data**^**b**^					
Creatinine (mg/dl)	1.03 ± 0.12	1.03 ± 0.12	1.02 ± 0.12	1.03 ± 0.12	0.741
Glomerular filtration rate (mL/min/1.73 m2)	79.8 ± 10.0	79.7 ± 9.8	80.0 ± 9.8	80.0 ± 10.0	0.748
**Nutrient Intake**^c^					
Energy(Kcal/d)	2410 (2374—2446)	2385 (2349—2420)	2376 (2341—2412)	2238 (2202—2274)	< 0.001
Carbohydrate(% of energy)	60.3 (59.9 – 60.9)	58.2 (57.8 – 58.6)	57.7 (57.3 –58.2)	56.9 (56.5 – 57.3)	< 0.001
Protein(% of energy)	15.1 (14.8 – 15.4)	14.6 (14.3 – 14.9)	14.4 (14.1 – 14.7)	13.9 (13.6 – 14.2)	< 0.001
Fat(% of energy)	29.9 (29.3 – 30.6)	30.7 (30.0 – 31.3)	30.7 (30.1 – 31.4)	31.5 (30.8 – 32.2)	0.015
**DIS components**^c^					
Leafy greens and Cruciferous vegetables (g/d)	41.5 (39.5 – 43.6)	28.0 (25.9 – 30.0)	21.3 (19.2 – 23.3)	15.0 (12.9 – 17.0)	< 0.001
Tomatoes(g/d)	163.4 (159.0 – 167.8)	104 (99.8 – 108.5)	74.2 (69.8 – 78.5)	47.1 (42.6 – 51.5)	< 0.001
Apples and berries(g/d)	161.1 (156.9 – 165.2)	92.4 (88.3 – 96.5)	60.0 (56.0—6401)	32.9 (28.8- 37.1)	< 0.001
Deep yellow or orange Vegetables and fruit(g/d)	108.5 (105.1—111.8)	61.5 (58.204—64.8)	41.9 (38.6—45.2)	24.6 (21.2—27.9)	< 0.001
Other fruits and real fruit juices(g/d)	440 (428—452)	302 (291—314)	221 (209—232)	139 (127—151)	< 0.001
Other vegetables(g/d)	222 (217—227)	168 (163—173)	138 (133—143)	98 (93—103)	< 0.001
Legumes(g/d)	38.7 (36.7—40.6)	35.9 (34.0—37.8)	35.6 (33.7—37.5)	28.5 (26.6—30.4)	< 0.001
Fish(g/d)	12.5 (11.6—13.5)	12.2 (11.2—13.1)	11.0 (10.1 – 12.0)	8.9 (7.9—9.9)	< 0.001
Poultry(g/d)	36.0 (34.4—37.6)	30.1 (28.6—31.7)	26.7 (25.1—28.2)	21.051 (19.5—22.6)	< 0.001
Red and organ meats(g/d)	35.4 (34.0—36.8)	36.7 (35.3—38.1)	38.1 (36.8—39.5)	38.0 (36.6—39.4)	< 0.001
Processed meats(g/week)	1.97 (0.32—4.92)	3.44 (0.81- 6.89)	3.94 (1.21- 8.87)	6.07 (2.95 – 16.61)	< 0.001
Added sugars(g/d)	80.9 (75.8—86.0)	86.0 (81.0—91.0)	92.7 (87.7—97.7)	90.7 (85.6—95.8)	0.008
High-fat dairy(g/d)	140 (132—148)	148 (140—156)	153 (145—160)	136 (128—144)	0.012
Low-fat dairy(g/d)	242 (233—251)	229 (220—238)	209 (200—218)	171 (161—180)	< 0.001
Coffee and tea(g/d)	762 (734—789)	648 (621—674)	584 (558—611.7)	467 (439—494)	< 0.001
Nuts(g/d)	12.5 (11.8—13.1)	8.4 (7.7 – 9.0)	6.5 (5.8—7.1)	4.2 (3.5—4.8)	< 0.001
Other fats(g/d)	23.0 (22.0—24.1)	27.3 (26.4—28.4)	28.2 (27.1—29.2	31.7 (30.7—32.8)	< 0.001
Refined grains and Starchy vegetables(g/d)	395 (384—405)	465 (455—475)	522 (512—532)	566 (555—576)	< 0.001

^a^ Data were represented as mean ± SD, or median (IQR 25–75) for continuous variables and percent for categorical variables. Chi-square and linear regression were used to test the trend of continuous and categorical variables across quartiles of the DIS (as the median value in each quartile), respectively

^b^Data were represented as mean ± SD computed using univariate analysis adjusted for age and sex across quartiles of DIS, and *P*-value of the univariate analysis was reported as P for trend

^c^Data were represented as mean (95%CI) computed using univariate analysis adjusted for age and sex across quartiles of DIS, and P-value of the univariate analysis was reported as P for trend

Table 3 indicates the findings on the HRs of CKD across quartiles of FBDI, DIS, and LIS. Based on the age and sex-adjusted model, the higher score of LIS (HR = 1.26; 95%CI: 1.06–1.49, P for trend: 0.011) and FBDI (HR = 1.18; 95%CI: 1.01–1.38, P for trend: 0.024) was associated with increment risk of CKD. However, no significant association was found between the higher score of DIS and the risk of developing CKD (HR = 1.03; 95%CI: 0.86–1.24, P for trend: 0.321). Also, in the multivariable-adjusted model, the positive relationship between LIS (OR = 1.28; 95% CI:1.07–1.55, P for trend:0.006) and FBDI (HR = 1.21;95%CI:1.03–1.42, P for trend:0.014) and risk of CKD was remained significant, while no significant association was observed between higher DIS score and CKD risk based on final model (HR = 1.03;95%CI:0.86–1.23, P for trend:0.524).

**Table 3:** The association between the inflammatory indices and incidence of chronic kidney disease

	Quartiles of Lifestyle and dietary inflammatory indices				P trend
	Q1	Q2	Q3	Q4	
**FBDI** score					
Median (interquartile)	-3.96 (-5.73, -2.72)	0.20 (-0.69, 1.08)	4.08 (2.94, 5.21)	10.69 (8.48, 14.98)	
Follow up period	7.68 ± 2.76	7.92 ± 2.61	7.72 ± 2.74	7.70 ± 2.77	
person-years	11,615	11,972	11,672	11,639	
Case/Total	273/1511	298/1511	316/1511	329/1511	
Incidence rate (10.000 person year)	228	254	271	283	
Model 1*	1.00 (Ref)	0.99 (0.84 – 1.16)	1.11 (0.94 – 1.29)	1.18 (1.01 – 1.38)	0.024
Model 2^†^	1.00 (Ref)	1.01 (0.85 – 1.18)	1.13 (0.96 – 1.32)	1.21 (1.03 – 1.42)	0.014
**DIS** score					
Median (interquartile)	-0.73 (-1.07, -0.50)	-0.14 (-0.25, -0.03)	0.24 (0.15, 0.35)	0.65 (0.50, 0.83)	
Follow up period	7.60 ± 2.79	7.67 ± 2.79	7.88 ± 2.64	7.89 ± 2.65	
person-years	11,485	11,575	11,912	11,915	
Case/Total	412/1511	198/1511	255/1511	351/1511	
Incidence rate (10.000 person year)	358	166	214	302	
Model 1*	1.00 (Ref)	1.07 (0.92 – 1.23)	0.85 (0.73 – 1.00)	1.03 (0.86 – 1.24)	0.321
Model 2^†^	1.00 (Ref)	1.09 (0.94– 1.26)	0.86 (0.73 – 1.01)	1.03 (0.86 – 1.23)	0.524
**LIS** score					
Median (interquartile)	-0.18 (-0.41, 0.00)	0.48 (0.48, 0.71)	0.89 (0.89, 0.89)	1.39 (1.16, 1.57)	
Follow up period	8.17 ± 2.52	7.63 ± 2.74	7.73 ± 2.85	7.40 ± 2.87	
person-years	14,756	1187	6180	10,002	
Case/Total	229//1806	350/1554	195/799	374/1350	
Incidence rate (10.000 person year)	155	294	315	373	
Model 1*	1.00 (Ref)	1.25 (1.05 – 1.47)	1.17 (0.97 – 1.42)	1.26 (1.06 – 1.49)	0.011
Model 2^‡^	1.00 (Ref)	1.27 (1.06 – 1.52)	1.26 (1.03 – 1.53)	1.28 (1.07 – 1.55)	0.006

^*^Model 1: adjusted for age and sex

^†^Model 2: additionally adjusted for model 1 and body mass index, smoking, physical activity, education level, energy intake, type 2 diabetes, and hypertension

^‡^Model 2: additionally adjusted for model 1 and education level and energy intake, type 2 diabetes, and hypertension

We also assessed the combined role of the inflammatory potential of diet and lifestyle, determined by DIS-LIS and FBDI-LIS indices, in predicting the risk of CKD and showed results in Fig. 2A-B. In the multivariable-adjusted model, the higher scores of DIS-LIS (HR = 1.26; 95%CI: 1.06–1.50, P for trend < 0.05) (Fig. 2 A) and FBDI-LIS (HR = 1.41; 95%CI: 1.17–1.70, P for trend < 0.05) (Fig. 2 B) were related to increased risk of CKD incidence.

Table 4 showed the results on the association of FBDI, DIS, LIS, FBDI-LIS, DIS-LIS scores (per each quartile increase) with risk of CKD using subgroup analysis based on various important variables including sex, smoking status, education status, diabetes, and hypertension. Our findings showed that the HRs reported from these subgroup analyses based on different variables classification are not remarkably different. Based on sex classification, in women, the association of LIS score (HR:1.06 vs.1.03), FBDI-LIS score (HR:1.10 vs. 1.08), and DIS-LIS score (HR:1.07 vs.1.05) with the risk of CKD were slightly stronger than men group. Also, in diabetic patients, the HR of CKD according to the FBDI score (1.16 vs.1.10), LIS score (1.07 vs.1.06), FBDI-LIS score (1.16 vs.1.10), and DIS-LIS score (1.10 vs.1.07) was higher than non-diabetic patients, however, due to the low sample size, the results were statistically insignificant in diabetic patients group. Also, in hypertensive patients, the HR of CKD was higher than in non-hypertensive subjects based on FBDI score (1.09 vs.1.06), LIS score (1.12 vs.1.03), FBDI-LIS score (1.20 vs.1.05), and DIS-LIS score (HR: 1.12 vs.1.04). In smokers group, the association of LIS score (HR: 1.07 vs.0.92), FBDI-LIS score (HR: 1.11 vs.0.99), DIS-LIS score (HR: 1.08 vs.0.88) with the risk of CKD was higher than non-smoked participants. Furthermore, in the current study, participants with higher educational level were slightly more at risk of CKD than those with a lower education level.

**Table 4:** Stratified analysis for the association between the inflammatory indices (per each quartile increase) and incidence of chronic kidney disease based on sex, diabetes, hypertension, smoking, educational level classification

Variables	Lifestyle and dietary inflammatory indices									
	FBDI score		DIS score		LIS score		FBDI-LIS score		DIS-LIS	
	HR (95% CI)	*P*-value	HR (95% CI)	*P*-value	HR (95% CI)	*P*-value	HR (95% CI)	*P*-value	HR (95% CI)	*P*-value
**Sex**										
*Male(n* = *2760)*	1.07 (0.99–1.17)	0.078	0.97 (0.88–1.05)	0.495	1.03 (0.94–1.12)	0.440	1.08 (0.99–1.19)	0.063	1.05 (0.96–1.14)	0.262
*Female(n* = *3284)*	1.06 (0.99–1.14)	0.082	0.98 (0.91–1.05)	0.601	1.06 (0.99–1.14)	0.070	1.10 (1.02–1.18)	0.013	1.07 (1.00–1.15)	0.049
**Diabetes**										
*Diabetic patients (n* = *430)*	1.16 (0.98–1.38)	0.079	0.97 (0.83–1.14)	0.767	1.07 (0.91–1.25)	0.378	1.16 (0.98–1.38)	0.079	1.10 (0.94–1.29)	0.206
*Non-diabetic adults (n* = *5614)*	1.10 (1.03–1.17)	0.001	0.97 (0.91–1.03)	0.357	1.06 (1.009 -1.13)	0.024	1.10 (1.03–1.17)	0.001	1.07 (1.01–1.13)	0.022
**Hypertension**										
*Hypertensive patients (n* = *852)*	1.09 (0.98–1.21)	0.086	0.99 (0.89–1.11)	0.951	1.12 (1.00–1.25)	0.049	1.20 (1.06–1.35)	0.003	1.12 (1.003–1.25)	0.045
*Non-hypertensive adults (n* = *5192)*	1.06 (0.99–1.13)	0.071	0.96 (0.90–1.03)	0.336	1.03 (0.96–1.09)	0.360	1.05 (0.99–1.12)	0.098	1.04 (0.97–1.10)	0.198
**Smoking status**										
*No-smokers(n* = *5320)*	1.10 (0.92–1.32)	0.269	0.86 (0.72–1.03)	0.109	0.92 (0.76–1.13)	0.460	0.99 (0.82–1.21)	0.972	0.88 (0.74–1.06)	0.193
*Smokers(n* = *724)*	1.07 (1.01–1.13)	0.019	0.98 (0.93–1.04)	0.667	1.07 (1.01–1.13)	0.018	1.11 (1.04–1.18)	0.001	1.08 (1.02–1.15)	0.005
**Education status**										
*Academic degree (n* = *1415)*	1.06 (0.94–1.20)	0.319	1.26 (0.82–1.94)	0.275	1.07 (0.94–1.22)	0.269	1.12 (0.98–1.28)	0.077	1.13 (0.99–1.29)	0.057
*Diploma and lower(n* = *4629)*	1.07 (1.01–1.14)	0.017	0.95 (0.78–1.17)	0.678	1.05 (0.99–1.12)	0.083	1.09 (1.02–1.16)	0.005	1.05 (0.99–1.11)	0.087

^*^Analyses adjusted for age, sex (except of sex category), body mass index, smoking (except of smoking category), physical activity, education level (except of education status), energy intake, type 2 diabetes (except of diabetes status), and hypertension (except of hypertension status)

## Discussion

The current study provided strong evidence that a lifestyle with a higher score of LIS was significantly associated with an increased risk of CKD, independent of major potential confounders in Tehranian adults. Also, a dietary pattern with a high score of FBDI was positively related to the risk of CKD in our study populations. However, no significant association was observed between a high score of DIS and CKD risk.

To the best of our knowledge, our study is the first to investigate the association of pro-inflammatory lifestyle and dietary pattern with the incidence of CKD in a longitudinal prospective analysis among adults. Our results are comparable with findings of previously published observational studies conducted on non-CKD subjects in which a pro-inflammatory lifestyle and dietary pattern with a high score of LIS, DIS, and FBDI can be associated with CKD-related disorders such as T2D and MetS or other nutrition-related chronic diseases [15–21]. Teymoori et al. have shown a positive relationship between a higher LIS score and incidence of T2D, but no significant association was observed between this inflammatory score and the T2D risk [20]. Based on the findings of Farhadnejad et al. study, there was a remarkable association between lifestyle and diet with high DIS and LIS scores and increased risk of MetS [17]. Also, prospective studies suggested that pro-inflammatory diets and lifestyles with higher LIS and DIS scores may be associated with colorectal cancer incidence [18, 19] and greater risk of all-cause, cancer- and CVD-specific mortality [16]. Furthermore, the Na et al. study showed that an increased FBDI score was related to higher odds of MetS [15].

Our results reported that individuals with high FBDI score adhered to the pro-inflammatory dietary pattern, which is characterized by various dietary determinants intakes that each of them may contribute to developing kidney dysfunction and increased CKD risk. This pro-inflammatory dietary pattern was defined by higher intakes of sweetened drinks, refined grain, and red meat and lower intakes of green vegetables, eggs, citrus fruits, red fruits, whole grain, legumes, and nuts. Therefore, it is expectable that high variation in intakes of the above-mentioned dietary factors can play an essential role in the risk of chronic diseases such as CKD through the effect on systemic inflammation; e.g., previous investigations have suggested that higher intakes of fruits, vegetables, and whole-grain or their bioactive compounds are associated with lower levels of inflammatory markers (such as CRP and ILs) [28–30], and were inversely associated with kidney dysfunction [31]. Also, anti-inflammatory healthy diet indices such as the Mediterranean diet and the Dietary Approaches to Stop Hypertension (DASH) diet focuses on higher consumption of legumes, nuts, whole grain, vegetables, and fruits, and lower consumption of red and processed meat, saturated fats, and simple sugar, have shown remarkable anti-inflammatory effects [32, 33] and protective role in the prevention of CKD [34, 35]. Furthermore, it has been reported that higher adherence to an unhealthy dietary pattern, characterized by higher intakes of red and processed meats, simple sugar, and foods with high saturated fats, is related to higher inflammatory indices, including CRP and IL-6 [36]. Thus, it is acceptable to declare that greater adherence to a high pro-inflammatory dietary pattern resulted in an increased risk of low-grade chronic inflammation and progression of renal dysfunction through-provoking in pro-inflammatory indices and reducing of anti-inflammatory markers.

In our study, the LIS index was introduced as a strong adverse predictor of kidney function in comparison to the DIS index; this finding is justifiable because of the inappropriate levels of each of the components of this index (elevated BMI, physical inactivity activity, and smoking) are considered as a main pro-inflammatory risk factor of the CKD risk, individually; therefore the cooperative contributions of these lifestyle-related factors to the inflammatory process have increased the risk of kidney dysfunction compared with DIS index. It has been revealed that adiposity is linked to the higher levels of various inflammatory indicators such as IL-6, TNF-α, and adipokines [37, 38] that, in this way, indirectly lead to disruption of kidney function [39]. Also, heavy smoking can have a nephrotoxic effect [40] via chronic effects on pro-inflammatory reactions, up-regulating inflammatory markers, increased oxidative stress, and glomerulosclerosis [41, 42]. Furthermore, previous reports have suggested that a sedentary lifestyle is associated with low‐grade inflammation, characterized by increased levels of inflammatory indicators such as hs-CRP and IL-6 [43]. Therefore, the cooperative contributions of main lifestyle factors including elevated BMI, heavy smoking, and low physical activity level to inflammation, indicated a significant association between LIS and CKD incidence in compared to the DIS index as a dietary index that only focused on inflammatory potential of diet.

DIS is a dietary inflammatory score based on various food groups that focus on a wide range of dietary foods, so the possible interaction of anti/pro-inflammatory food components of this dietary inflammatory score can attenuate its prediction abilities for CKD risk. Also, based on our findings, the individuals' intakes for food components of DIS were close to each other and did not have high dispersion; therefore, we observed a narrow range for the DIS score in participants, which reduced the predictive power of this inflammatory index in CKD risk. Furthermore, the DIS score has been developed and validated in a different population, which may have differences in general characteristics such as lifestyle, dietary habits, and genetic background with our people, therefore more studies are needed to assess the applicability of the DIS index for prediction of CKD in our population. Finally, in our study, one of the reasons for the greater power of the LIS index than DIS in predicting the risk of CKD is the cooperative contributions of major lifestyle-related LIS components, including BMI, physical activity, and smoking to inflammation that revealed a greater association with CKD in compared to the DIS that focused only on dietary components. Therefore, the inflammatory conditions created by LIS components in subjects in the fourth quartile of LIS can lead participants much more prone to increased risk of CKD.

The strengths of the current study include its population-based prospective setting, relatively large sample size, and long follow-up time duration. Also, our study is the first survey that investigates the relationship between LIS, FBDI, and DIS and CKD incidence risk. Furthermore, we used valid and reliable questionnaires to collect participants' dietary intakes and physical activity data. However, our study had some limitations. Some items were excluded in the calculation of DIS and LIS; the final scores were computed based on 18 instead of 19 items for DIS due to the lack of data in our dataset on nutritional supplement intakes. Also, we used 3 instead of 4 items for LIS; because of religious and legal restrictions in the Iranians, alcohol is not consumed, or its consumption is not reported, so we did not consider alcohol consumption to compute LIS. Similar to other observational studies, FFQ was used for the nutritional assessment of participants, which measurement error is expected. Finally, there is possible residual confounding which we cannot exclude due to unknown or unmeasured factors.

## Conclusion

Our population-based cohort study suggested that a higher inflammatory potential of diet and lifestyle, determined by the higher score of FBDI and LIS, were associated with increased incidence of CKD in Iranian adults; however, no significant association was observed between the higher DIS score and risk of CKD. Further epidemiological studies are recommended to address the possible inflammatory effects of lifestyle and dietary pattern and their combinations in CKD development and its potential mechanisms.

## Data Availability

The datasets analyzed in the current study are available from the corresponding author on reasonable request.

## Abbreviations

CKD: Chronic kidney disease
CIs: Confidence intervals
CVD: Cardiovascular disease
DBP: Diastolic blood pressure
DIS: Diet inflammation score
EPI: Epidemiology Collaboration
FBDI: Food-based dietary inflammatory potential
FCT: Food Composition Table
FFQ: Food frequency questionnaire
FPG: Fasting blood glucose
GFR: Glomerular filtration rate
HRs: Hazard ratios
HTN: Hypertension
LIS: Lifestyle inflammation score
MAQ: Modifiable activity questionnaire
Met.h.wk: Metabolic equivalent/hours per week
MetS: Metabolic syndrome
SBP: Systolic blood pressure
T2D: Type 2 diabetes
TLGS: Tehran Lipid and Glucose Study
USDA: United States Department of Agriculture

## References

[1] Cockwell P, Fisher L-A (2020). The global burden of chronic kidney disease. Lancet.

[2] Vallianou NG, Mitesh S, Gkogkou A, Geladari E (2019). Chronic kidney disease and cardiovascular disease: is there any relationship?. Curr Cardiol Rev.

[3] Zhang Q, Ma Y, Lin F, Zhao J, Xiong J (2020). Frailty and mortality among patients with chronic kidney disease and end-stage renal disease: a systematic review and meta-analysis. Int Urol Nephrol.

[4] Farhadnejad H, Asghari G, Emamat H, Mirmiran P, Azizi F (2019). Low-carbohydrate high-protein diet is associated with increased risk of incident chronic kidney diseases among Tehranian adults. J Ren Nutr.

[5] Wakasugi M, Kazama JJ, Yamamoto S, Kawamura K, Narita I (2013). A combination of healthy lifestyle factors is associated with a decreased incidence of chronic kidney disease: a population-based cohort study. Hypertens Res.

[6] Kanzaki G, Tsuboi N, Haruhara K, Koike K, Ogura M, Shimizu A, Yokoo T (2015). Factors associated with a vicious cycle involving a low nephron number, hypertension and chronic kidney disease. Hypertens Res.

[7] Tinti F, Lai S, Noce A, Rotondi S, Marrone G, Mazzaferro S, Di Daniele N, Mitterhofer AP: Chronic kidney disease as a systemic inflammatory syndrome: update on mechanisms involved and potential treatment. Life (Basel) 2021, 11:419. 10.3390/life11050419 PMC8147921 34063052

[8] Dai L, Golembiewska E, Lindholm B, Stenvinkel P (2017). End-stage renal disease, inflammation and cardiovascular outcomes. Contrib Nephrol.

[9] Kolb H, Mandrup-Poulsen T (2010). The global diabetes epidemic as a consequence of lifestyle-induced low-grade inflammation. Diabetologia.

[10] Galland L (2010). Diet and inflammation. Nutr Clin Pract.

[11] Petersen AM, Pedersen BK (1985). The anti-inflammatory effect of exercise. J Appl Physiol.

[12] Attard R, Dingli P, Doggen CJ, Cassar K, Farrugia R, Wettinger SBJOH: The impact of passive and active smoking on inflammation, lipid profile and the risk of myocardial infarction. 2017, 4. 10.1136/openhrt-2017-000620 PMC5574419 28878948

[13] Bianchi VE (2018). Weight loss is a critical factor to reduce inflammation. Clin Nutr ESPEN.

[14] Byrd DA, Judd SE, Flanders WD, Hartman TJ, Fedirko V, Bostick RM (2019). Development and Validation of Novel Dietary and Lifestyle Inflammation Scores. J Nutr.

[15] Na W, Yu TY, Sohn C (2019). Development of a food-based index of dietary inflammatory potential for Koreans and its relationship with metabolic syndrome. Nurs Res Pract.

[16] Li Z, Gao Y, Byrd DA, Gibbs DC, Prizment AE, Lazovich D, Bostick RM (2021). Novel Dietary and Lifestyle Inflammation Scores Directly Associated with All-Cause, All-Cancer, and All-Cardiovascular Disease Mortality Risks Among Women. J Nutr.

[17] Farhadnejad H, Parastouei K, Rostami H, Mirmiran P, Azizi FJD, Syndrome M (2021). Dietary and lifestyle inflammatory scores are associated with increased risk of metabolic syndrome in Iranian adults.

[18] Byrd DA, Judd SE, Flanders WD, Hartman TJ, Fedirko V, Agurs-Collins T, Bostick RM: Associations of novel dietary and lifestyle inflammation scores with incident colorectal cancer in the NIH-AARP diet and health study JNCI Cancer Spectrum 2020, 4:pkaa009. 10.1093/jncics/pkaa009 PMC7236782 32455332

[19] Byrd DA, Judd S, Flanders WD, Hartman TJ, Fedirko V (2020). Bostick RMJCE. Biomarkers P: Associations of novel dietary and lifestyle inflammation scores with incident, sporadic colorectal adenoma.

[20] Teymoori F, Farhadnejad H, Mokhtari E, Sohouli MH, Moslehi N, Mirmiran P, Azizi F (2021). Dietary and lifestyle inflammatory scores and risk of incident diabetes: a prospective cohort among participants of Tehran lipid and glucose study. BMC Public Health.

[21] Yoon HS, Shon J, Park YJ (2022). Effects of Korean Food-based Dietary Inflammatory Index Potential on the incidence of diabetes and HbA1c level in Korean adults aged 40 years and older. J Nutr Health.

[22] Azizi F, Ghanbarian A, Momenan AA, Hadaegh F, Mirmiran P, Hedayati M, Mehrabi Y, Zahedi-Asl S (2009). Prevention of non-communicable disease in a population in nutrition transition: Tehran Lipid and Glucose Study phase II. Trials.

[23] Momenan AA, Delshad M, Sarbazi N (2012). Rezaei_Ghaleh N, Ghanbarian A, Azizi F: Reliability and validity of the Modifiable Activity Questionnaire (MAQ) in an Iranian urban adult population. Arch Iran Med.

[24] James PA, Oparil S, Carter BL, Cushman WC, Dennison-Himmelfarb C, Handler J, Lackland DT, LeFevre ML, MacKenzie TD, Ogedegbe O (2014). 2014 evidence-based guideline for the management of high blood pressure in adults: report from the panel members appointed to the Eighth Joint National Committee (JNC 8). JAMA.

[25] Association AD (2003). Report of the expert committee on the diagnosis and classification of diabetes mellitus. Diabetes Care.

[26] Levey AS, Stevens LA, Schmid CH, Zhang YL, Castro AF, Feldman HI, Kusek JW, Eggers P, Van Lente F, Greene T (2009). A new equation to estimate glomerular filtration rate. Ann Intern Med.

[27] Mirmiran P, Esfahani FH, Mehrabi Y, Hedayati M, Azizi F (2010). Reliability and relative validity of an FFQ for nutrients in the Tehran lipid and glucose study. Public Health Nutr.

[28] Byrd D, Holmes A, Judd S, Flanders WD, Bostick RM: Associations of whole food and lifestyle-based inflammation scores with all-cause, cancer-and cardiovascular disease-specific mortality. AACR; 2017.

[29] Xu Y, Wan Q, Feng J, Du L, Li K, Zhou Y: Whole grain diet reduces systemic inflammation: A meta-analysis of 9 randomized trials. Medicine 2018, 97. 10.1097/MD.0000000000012995 PMC6221555 30412134

[30] Zhu F, Du B, Xu B (2018). Anti-inflammatory effects of phytochemicals from fruits, vegetables, and food legumes: A review. Crit Rev Food Sci Nutr.

[31] Nettleton JA, Steffen LM, Palmas W, Burke GL, Jacobs DR (2008). Associations between microalbuminuria and animal foods, plant foods, and dietary patterns in the Multiethnic Study of Atherosclerosis. Am J Clin Nutr.

[32] Bonaccio M, Pounis G, Cerletti C, Donati MB, Iacoviello L, de Gaetano G (2017). Mediterranean diet, dietary polyphenols and low grade inflammation: results from the MOLI-SANI study. Br J Clin Pharmacol.

[33] Soltani S, Chitsazi MJ, Salehi-Abargouei A (2018). The effect of dietary approaches to stop hypertension (DASH) on serum inflammatory markers: a systematic review and meta-analysis of randomized trials. Clin Nutr.

[34] Asghari G, Farhadnejad H, Mirmiran P, Dizavi A, Yuzbashian E, Azizi F (2017). Adherence to the Mediterranean diet is associated with reduced risk of incident chronic kidney diseases among Tehranian adults. Hypertens Res.

[35] Asghari G, Yuzbashian E, Mirmiran P, Azizi F: The association between Dietary Approaches to Stop Hypertension and incidence of chronic kidney disease in adults: the Tehran Lipid and Glucose Study. Nephrology Dialysis Transplantation 2017, 32:ii224-ii230. 10.1093/ndt/gfw273 28201810

[36] Bawaked RA, Schröder H, Ribas-Barba L, Izquierdo-Pulido M, Pérez-Rodrigo C, Fíto M, Serra-Majem L (2017). Association of diet quality with dietary inflammatory potential in youth. Food Nutr Res.

[37] Lyon CJ, Law RE, Hsueh WA (2003). Minireview: adiposity, inflammation, and atherogenesis. Endocrinology.

[38] Elks CM, Francis J (2010). Central adiposity, systemic inflammation, and the metabolic syndrome. Curr Hypertens Rep.

[39] Mihai S, Codrici E, Popescu ID, Enciu A-M, Albulescu L, Necula LG, Mambet C, Anton G, Tanase C: Inflammation-related mechanisms in chronic kidney disease prediction, progression, and outcome. Journal of Immunology Research 2018, 2018. 10.1155/2018/2180373 PMC6146775 30271792

[40] Xia J, Wang L, Ma Z, Zhong L, Wang Y, Gao Y, He L, Su X (2017). Cigarette smoking and chronic kidney disease in the general population: a systematic review and meta-analysis of prospective cohort studies. Nephrol Dial Transplant.

[41] Stadler M, Tomann L, Storka A, Wolzt M, Peric S, Bieglmayer C, Pacini G, Dickson SL, Brath H, Bech P (2014). Effects of smoking cessation on β-cell function, insulin sensitivity, body weight, and appetite. Eur J Endocrinol.

[42] Anan F, Takahashi N, Shinohara T, Nakagawa M, Masaki T, Katsuragi I, Tanaka K, Kakuma T, Yonemochi H, Eshima N (2006). Smoking is associated with insulin resistance and cardiovascular autonomic dysfunction in type 2 diabetic patients. Eur J Clin Invest.

[43] Fischer C, Berntsen A, Perstrup L, Eskildsen P, Pedersen B (2007). Plasma levels of interleukin-6 and C-reactive protein are associated with physical inactivity independent of obesity. Scand J Med Sci Sports.

