# Correction: High dietary and lifestyle inflammatory scores are associated with increased risk of chronic kidney disease in Iranian adults

**DOI:** 10.1186/s12937-023-00839-8

**Published:** 2023-01-12

**Authors:** Hossein Farhadnejad, Farshad Teymoori, Mitra Kazemi Jahromi, Ebrahim Mokhtari, Golaleh Asghari, Parvin Mirmiran, Fereidoun Azizi

**Affiliations:** 1 Nutrition and Endocrine Research Center, Research Institute for Endocrine Sciences, Shahid Beheshti University of Medical Sciences, Tehran, Iran; 2 Department of Clinical Nutrition and Dietetics, Faculty of Nutrition Sciences and Food Technology, National Nutrition and Food Technology Research Institute, Shahid Beheshti University of Medical Sciences, Tehran, Iran; 3 Department of Nutrition, School of Public Health, University of Medical Sciences, Tehran, Iran; 4 Endocrinology and Metabolism Research Center, Hormozgan University of Medical Sciences, Bandar Abbas, Iran; 5 Endocrine Research Center, Research Institute for Endocrine Sciences, Shahid Beheshti University of Medical Sciences, Tehran, Iran

**Correction: Nutr J 22, 1 (2023)**

**https://doi.org/10.1186/s12937-023-00835-y**

Following the publication of the original article [1], the author reported that the co-corresponding author was not captured. The corresponding authors are updated in this correction article and the original article has been updated.
